# High-Resolution Monitoring of Scour Using a Novel Fiber-Optic Distributed Temperature Sensing Device: A Proof-of-Concept Laboratory Study

**DOI:** 10.3390/s23073758

**Published:** 2023-04-05

**Authors:** Rebecca Hatley, Mahmoud Shehata, Chadi Sayde, Celso Castro-Bolinaga

**Affiliations:** Department of Biological and Agricultural Engineering, NC State University, Raleigh, NC 27695, USA

**Keywords:** monitoring, distributed temperature sensing, water depth sensing, scour, sediment transport

## Abstract

Scour events can severely change the characteristics of streams and impose detrimental hazards on any structures built on them. The development of robust and accurate devices to monitor scour is therefore essential for studying and developing mitigation strategies for these adverse consequences. This technical note introduces a novel scour-monitoring device that utilizes new advances in the fiber-optic distributed temperature sensing (FO-DTS) technology. The novel FO-DTS scour-monitoring device utilizes the differential thermal responses of sediment, water, and air media to a heating event to accurately identify the locations of the interfaces between them. The performance of the device was tested in a laboratory flume under flow conditions with water velocities ranging from 0 m/s to 0.16 m/s. In addition, the effect of the measurement duration on the device’s measurement accuracy was also investigated. The FO-DTS scour-monitoring device managed to detect the sediment–water and water–air interfaces with average absolute errors of 1.60 cm and 0.63 cm, respectively. A measurement duration of fewer than 238 s was sufficient to obtain stable measurements of the locations of the sediment–water and water–air interfaces for all the tested flow conditions.

## 1. Introduction

Scour refers to the removal of bed sediment around riverine infrastructure (e.g., bridge foundations) due to the erosive action of flowing water [1]. Despite major advances in understanding this phenomenon, the scour-driven failure of riverine infrastructure still represents major technical, economical, and societal challenges [2]. In the United States, there are roughly 500,000 bridges constructed over waterways [1], and among these, more than 20,000 bridges are currently at risk of being submerged or experiencing significant structural damage due to scour during extreme storm events [3]. Scour is also the leading cause of bridge failure, accounting for almost 60% of all failures [4,5] and for an estimated annual cost of approximately 30 million USD [6]. The accurate measurements of scour depths are therefore of critical importance for the design and construction of resilient riverine infrastructure, particularly in the context of climate change-induced fluctuations in rivers’ water-sediment regimes [7].

Many techniques have been developed to monitor scour, including those that use buried rods or float-out devices [4,8,9], electromagnetic or sound pulses [10,11,12], and strain mechanical devices [13]. Tonina et al. [14] and DeWeese et al. [15] developed and tested a scour-monitoring device that consists of several temperature sensors scattered along a tube, which is intended to be partially buried in the bed sediment. Changes in the amplitudes and phase shifts among the temperature time series, recorded at different depths, were used to identify the location of the interface between the sediment and water by solving a simplified heat transport equation. Several studies also investigated the possibility of using the capabilities of fiber Bragg grating (FBG) or the ultra-weak fiber Bragg grating (UWFBG) technologies for detecting vibrations or strain exerted upon fiber-optic (FO) cables to build scour detection sensors [9,13,16,17,18,19,20]. However, the main obstacle hindering the practical application of those sensors is the thin and fragile nature of the FO used in the FBG and the UWFBG technologies, which makes them susceptible to being damaged during extreme flooding events, when maximum scour typically develops [16,18,20].

The main limitations of all the aforementioned scour-monitoring techniques can be summarized by one or more of the following [15,16,21]: (1) inability to continuously monitor scour during flood events; (2) high-cost, low-durability, and/or high-maintenance requirements; (3) measurement errors due to the influence of environmental factors, such as variation in water temperature or water temperature stratification; and (4) low temporal and/or vertical scour-detecting resolutions. Hence, the development of a high-resolution, robust, and durable scour-monitoring device is highly required.

New advancements in the distributed temperature sensing (DTS) technology made it possible to measure temperature along a FO cable extending over several kilometers with spatial and temporal resolutions of up to 0.125 m and 1 s, respectively, with an accuracy that can reach 0.01 °C [22,23]. The fundamental principle behind the DTS technology involves using the backscattering of a laser pulse transmitted through a FO cable to measure temperatures. This is achieved by analyzing the intensities and detection times of the Stokes and anti-Stokes Raman backscattering signals, from which the temperature at the location along the FO cable initiating these particular scatterings can be estimated. The exact location of the temperature measurements along the FO cable can be determined by comparing the detection times of the Raman scatterings with the speed of light [24,25]. The unprecedented high-resolution temperature measurements obtained over large distances using the DTS technology were utilized to monitor and study different environmental and hydrological parameters, including but not limited to soil moisture content [26,27,28,29,30,31], groundwater upwelling [32], seepage rate in losing streams [33], wind speed, and temperature fluxes [22,34,35].

The DTS technology also has the potential to measure sediment scour in riverine conditions. The principle behind this type of measurement is that if an installed FO cable in a river is heated using a metallic component in its composition, the increase in temperature observed using the DTS over the length of the FO cable would depend on the thermal properties of the surrounding material (e.g., sediment, water, or air) and the strength of any existent convective cooling. The changes in the thermal and convective cooling properties observed across the sediment–water (S-W) or the water–air (W-A) interfaces will lead to anomalies in the observed temperature-increase (ΔT) profiles, which can then be used to detect their locations. Zhang and Zhao [36] used a heated FO cable to measure the S-W interface in a bucket filled with sediment and standing water for developing a subsea pipeline scour-monitoring system. They concluded that the FO cable was able to estimate the S-W interface with an error of less than 0.16 m in the tested conditions. Although promising, the Zhang and Zhao [36] tested conditions that are not representative of scour phenomena, in which sediment is rapidly transported by flowing water around bridge piers.

The objective of this study was to introduce a novel FO-DTS scour-monitoring device capable of accurately tracking changes in the S-W and W-A interfaces at high spatial and temporal resolution. A proof-of-concept laboratory study was conducted to test the performance of the novel device under different conditions, ranging from standing to flowing water. The influence of the measurement duration on the accuracy of the detected S-W and W-A interfaces was also investigated.

## 2. Materials and Methods

### 2.1. Fiber-Optic-Distributed Temperature Sensing (FO-DTS) Device

**Figure 1:**
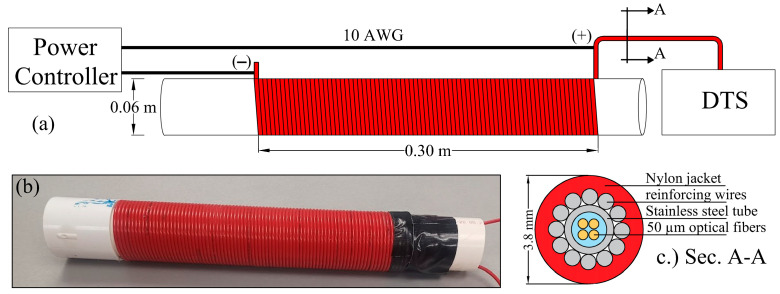
The FO-DTS scour-monitoring device: (**a**) schematic diagram of the constructed FO-DTS device, (**b**) photo of the actual device, and (**c**) cross-section of the used FO cable.

The FO-DTS scour-monitoring device was constructed by tightly wrapping a BRUsens^®^ FO cable (Brugg cables, Brugg, Switzerland) around a 0.06 m diameter pipe to reach a total vertical length of 0.3 m (Figure 1). The used FO cable has a total diameter of 3.8 mm and consists of 50 µm optical fibers encased within a gel-filled stainless-steel tube (inner diameter 1.07 mm and outer diameter 1.3 mm) surrounded by 0.42 mm diameter stainless steel reinforcing wires and covered by a 0.83 mm nylon jacket. The selection of this particular FO cable was based on its durability, ruggedness, and ability to resist various stresses. Previous studies have confirmed these characteristics, as the cable was successfully installed in soil over large distances (several hundred meters) using a plow without sustaining any damage or additional non-uniform signal losses [27,29]. Thus, the FO cable was deemed suitable for enduring the stresses that the FO-DTS scour-monitoring device could encounter during field deployments, especially from the impact of fast-moving debris under severe flooding conditions. Additionally, this FO cable is suitable for active heating as the stainless steel tube and wires integrated within it can be utilized as a heating element. In this study, the heating of the FO cable was performed by connecting the metallic component of the FO cable to a BK Precision 9205 DC power supply (B&K Precision Corp., Yorba Linda, CA, USA).

A Silixa XT^®^ (Silixa Ltd., Borehamwood, UK) DTS system with sampling and temporal resolutions of 0.25 m and 5 s was used to monitor the temperature over the whole length of the FO cable. The default internal calibration algorithm was used to generate the temperature profiles as the device only uses the locations of the abrupt temperature variations to detect the S-W or W-A interfaces and does not need accurate absolute temperature measurements.

### 2.2. Experimental Flume Characteristics

**Figure 2:**
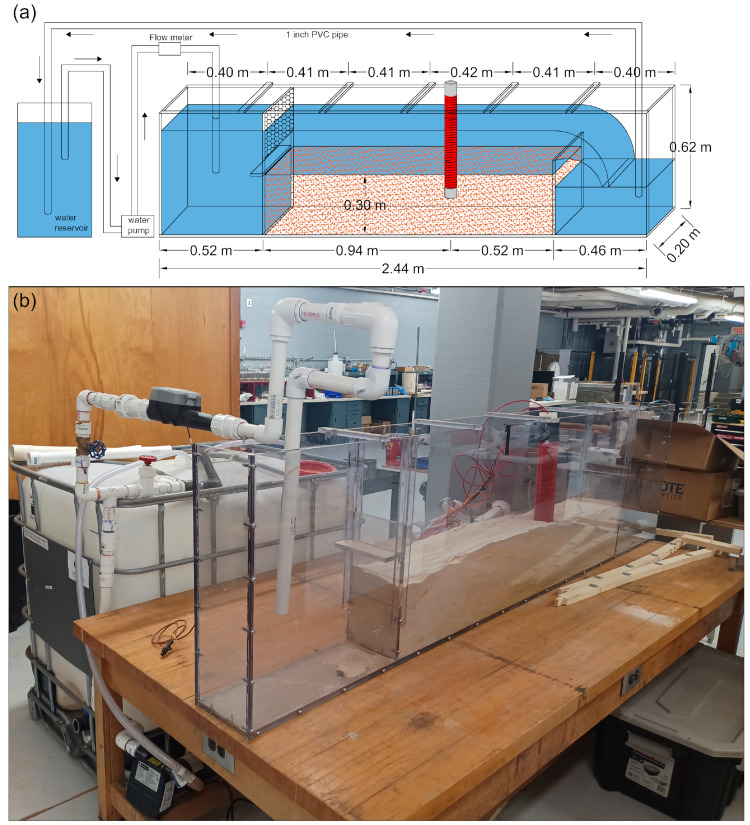
The used flume setup: (**a**) a schematic diagram of the flume setup showing its dimensions in meters and (**b**) the actual setup used to test the performance of the FO-DTS scour-monitoring device.

The performance of the FO-DTS scour-monitoring device was tested under different flow conditions inside a 2.44 m long by 0.2 m wide by 0.6 m deep flume (Figure 2). The testing section of the flume was filled with purified fine sand with a median grain size (denoted as D_50_ and defined as the diameter for which 50% of the bed material is finer by weight) of 0.30 mm and silicon dioxide content >99% (Glassil 530, Unimin Corporation-Marston, NC, USA), which was used to represent the bed sediment in this study. The FO-DTS scour-monitoring device was placed vertically in the flume so that part of its FO cable was buried under the sand, part of it was exposed directly to water, and the remaining section was exposed to the air. Water was pumped out of a reservoir into the inlet chamber of the flume through a series of 1-inch PVC pipes and valves equipped with a Dynasonics U500w Ultrasonic Flow Meter (Dynasonics, Burlington, VT, USA), which was used to measure the discharge passing through the flume. Water then flowed over the testing section, ultimately reaching the outlet chamber before it was recirculated back to the reservoir. L-shaped dividers were used to adjust the height of water flowing from the inlet chamber. The height of the outlet was also controlled. The water stage was measured by recording the depth of water over the testing section. The measured water discharge and stages were used to estimate the range of the water velocity running through the flume testing area.

### 2.3. FO-DTS Scour-Monitoring Device Testing Protocol

The FO-DTS scour-monitoring device was tested under three different flow conditions: (1) no-flow (standing water with a depth of 9 cm above the sand surface), (2) low-flow (water velocity ranging from 2.36 cm/s to 3.15 cm/s), and (3) high-flow (water velocity ranging from 8.85 cm/s to 15.92 cm/s). Three replicates were performed for each of the tested flow conditions. Before the start of each testing replicate, the bed was smoothed and leveled to have a uniform sand thickness of approximately 0.30 m, and the water was run through the flume for at least 30 min to reach thermal and hydraulic equilibrium. For each replicate, the FO cable was then heated using a heat pulse, which was achieved by passing a current of 7 amperes (20.3 W/m) through the FO cable’s metallic components over a duration ranging from 253 s to 306 s. This variation in the heat pulse duration was due to the manual control of the heat pulse application. The S-W and W-A interfaces for each replicate were estimated using the data from the first 250 s from the heat pulse start to remove any influence the variation in the heating duration might have over the results.

The ΔT profiles induced by the heat pulse application along the scour-monitoring device were used to estimate the S-W and W-A interfaces to eliminate the influence of the ambient temperature over the results. In this study, the ambient temperature was estimated as the mean temperature profile observed over the last 15 s before the start of the heat pulse. The temperature increase (ΔT) profiles along the scour-monitoring device due to the heat pulse application were estimated by subtracting the ambient temperature profile from the observed absolute temperature profiles.

### 2.4. Interface Detection Algorithm

The thermal and convection properties of the sediment, water, and air media control the temperature increase observed in them due to the applied heat pulse. Hence, it is expected to observe the maximum and minimum temperature increase in the sections exposed to air and water, respectively, with the temperature increase in the sediment between these two values. Therefore, significant increases in the temperature gradient magnitude will be observed at the locations of the interfaces between the three different media and can be used to detect them. 

**Figure 3:**
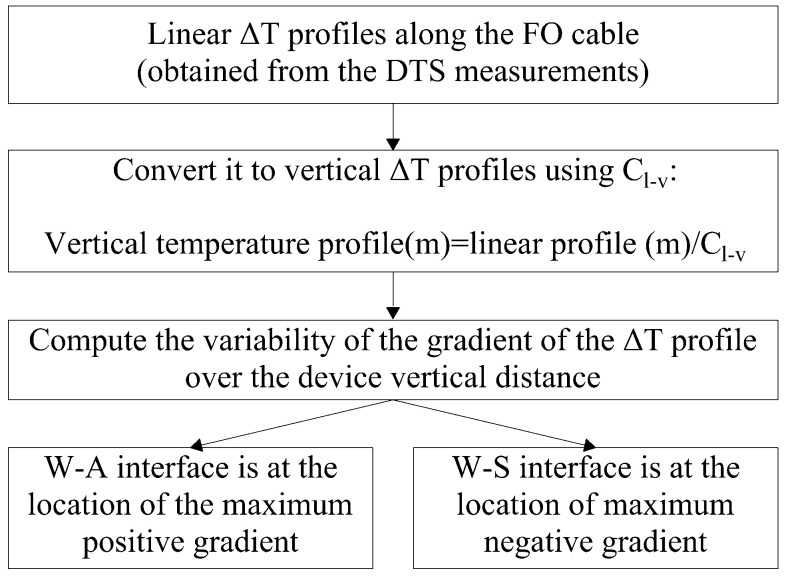
Flow chart of the algorithm used to detect the S-W and W-A interfaces.

An algorithm was created to automatically identify the location of the W-A and S-W interfaces. A flow chart of the scour detection algorithm is shown in Figure 3. The input to the algorithm was the vertical profile of the average ΔT obtained over the heat pulse duration of 600 s. The vertical profile was obtained from the linear ΔT profile observed along the FO cable by dividing its distance by a conversion coefficient $C_{l-v}$, which was estimated from the geometric relationship between the outer diameter of the PVC pipe ($r_{PVC}$) and the diameter of the FO cable ($r_{FO\;}$= 1.88 cm), according to Equation (1).

(1) $$C_{l-v}=\pi (r_{PVC}+r_{FO})/\;r_{FO}$$

Performing this calculation using the dimensions of the used FO-DTS scour-monitoring device showed that every 1 m on the vertical profile is represented by 54 m of linear distance measured along the FO cable. The vertical distance was referenced relative to the bottom end of the device located inside the sediment. Then, the variability of the gradient of the ΔT signal over the device’s vertical distance was calculated and was used to detect the locations of the W-A and S-W interfaces. The location of the W-A interface is identified as the location of the maximum positive ΔT gradient due to the abrupt increase in the recorded FO cable thermal response as it transitions from being surrounded by water to air. On the contrary, the S-W interface is detected by detecting the location of the maximum negative ΔT gradient as the thermal response of the cable abruptly decreases when transitioning from being surrounded by sediment to water. The results of the algorithm were compared against the observed locations of the interfaces, which were measured using a measuring tape before starting the heating for each replicate.

### 2.5. Determination of the Effect of the Measurement Duration 

Minimizing the duration of the measurement while maintaining appropriate measurement accuracy is important to allow higher frequency readings of the S-W and W-A interfaces. Thus, the performance of the FO-DTS scour-monitoring device in terms of detecting the location of the interfaces was evaluated for measurement durations ranging from 5 s to 400 s with 5 s increments (the DTS measuring interval). This was achieved by averaging the DTS measurements observed over the required measurement duration, starting from the initiation of the FO heating and using the averaged signal to detect the S-W and W-A interfaces. 

## 3. Results and Discussion

**Figure 4:**
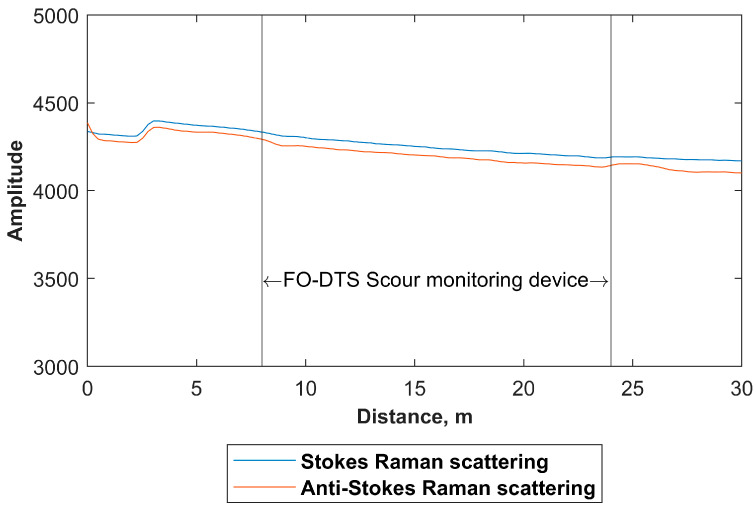
Example of the Stokes and anti-Stokes Raman scattering signals that were observed along the FO-DTS scour-monitoring device in a single measurement over a five second period before the start of the active heating.

The raw Stokes and anti-Stokes Raman scattering signals observed along the FO cable were examined to assess whether excessive and non-uniform losses developed in the FO cable from twining it around the PVC pipe. An example of the raw Stokes and anti-Stokes Raman scattering signals is shown in Figure 4, which illustrates that twining the FO cable around the PVC pipe did not result in any additional or sudden losses along the FO cable besides the consistent signal attenuation typically observed in the DTS applications. Therefore, this supports that the size of the used PVC pipe (diameter 0.06 m) was sufficient to build a reliable FO-DTS scour-monitoring device using BRUsens^®^ FO cable. Furthermore, the properties of the signals observed along the FO-DTS scour-monitoring device, coupled with the spatial coverage capabilities common in DTS units, indicate the potential to increase the length of the FO-DTS scour-monitoring device in field applications if necessary and that multiple devices can also be attached in a series to measure scour at various locations (e.g., multiple piers and abutments of a bridge).

**Figure 5:**
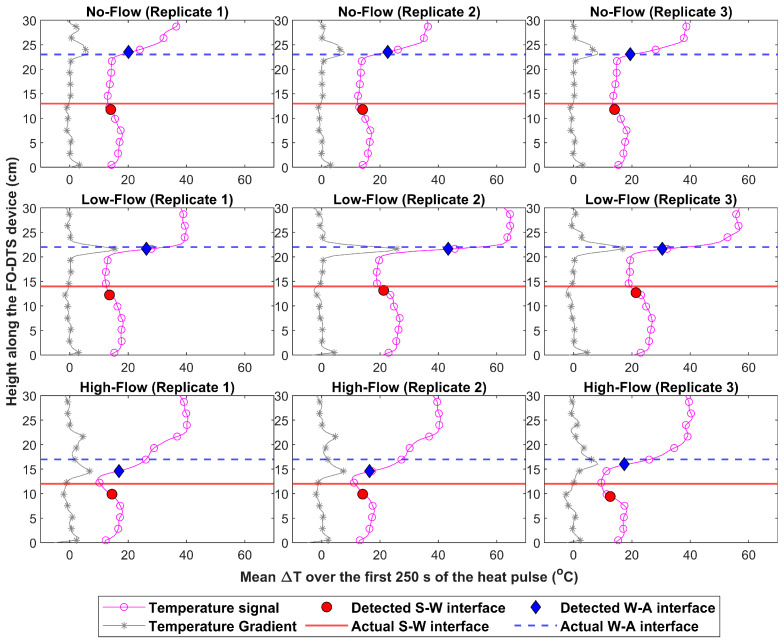
The mean temperature-increase profiles for the different testing conditions and a comparison between the detected locations of the interfaces versus their actual locations. The markers used to distinguish the ΔT and the gradient signals are added at every five measurements for clarity purposes.

The FO-DTS scour-monitoring device managed to provide accurate estimates of the locations of the S-W and the W-A interfaces (see Figure 5 and Table 1). No significant scour was observed in all the performed tests as the used flume was not capable of generating sufficient flow velocities to trigger scour formation. Sediment movement was only observed in the high-flow tests and resulted only in minor variations (<1 cm) in the locations of the S-W interface over the duration of these three tests. The manual measurements of the actual locations of the S-W interface in the high-flow tests did not capture the influence of the minor observed sediment movement as these measurements were performed before the beginning of the tests. Therefore, it should be noted that the slight differences in the measurements of the actual S-W interface locations observed in Figure 5 across the various tested velocity ranges were merely caused by variations in the level at which the sand was smoothed before each test, rather than by sediment movement or scour formation. 

For the no-flow testing conditions, the mean and standard deviation of the errors in the S-W and the W-A interfaces were −1.23 ± 0.00 cm and 0.38 ± 0.27 cm, respectively. Similar accuracy was achieved in the low-flow testing conditions where the mean error in the S-W and the W-A interfaces were −1.29 ± 0.47 cm and −0.35 ± 0.00 cm, respectively. On the other hand, relatively higher errors were observed in the high-flow tests with means of –2.27 ± 0.27 cm for the S-W interface and −1.94 ± 0.82 cm for the W-A interface. The relatively higher errors observed in the high-flow tests could be attributed to the slight sediment movement developing around the FO-DTS scour-monitoring device over time during these tests, which was not captured by the actual measurements of the S-W interface locations taken before the start of the heat application. Nevertheless, the results demonstrate that the FO-DTS scour-monitoring device has sufficient accuracy for most practical applications. 

**Figure 6:**
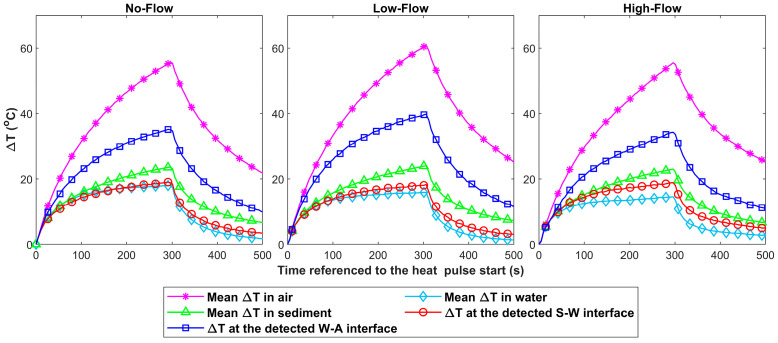
Examples of the  ΔT observed during and after the application of the heat pulse at the detected S-W and W-A interfaces and the mean ΔT observed in the device sections surrounded by sediment, water, and air. The markers used to distinguish the different ΔT signals were added every five measurements for clarity purposes.

For all the tested conditions, the highest and lowest rates by which ΔT increased during the heat pulse were observed as expected in the device sections surrounded by air and water, respectively, and an intermediate rate of ΔT increase was observed in the section surrounded by sediment (see Figure 6). In addition, it was noticed in all the tested conditions that the ΔT in the section surrounded by water started to drop rapidly once the heating stopped and almost reached an ambient temperature after approximately 200 s from the end of the heat pulse. This fast cooling rate of the water section highlights the potential of the FO-DTS scour-monitoring device to measure active scour and water depth with high temporal resolution. For instance, when the FO-DTS scour-monitoring device is subject to active scour conditions, the scour-exposed depth along the device was expected to cool rapidly after heating was halted compared to the section that remained buried in the sediments. This thermal behavior of the submerged section of the device was expected to allow frequent consecutive heat pulses, which increase the temporal resolution of scour detection of the device. A similar effect was expected in the case of water surface fluctuations.

**Figure 7:**
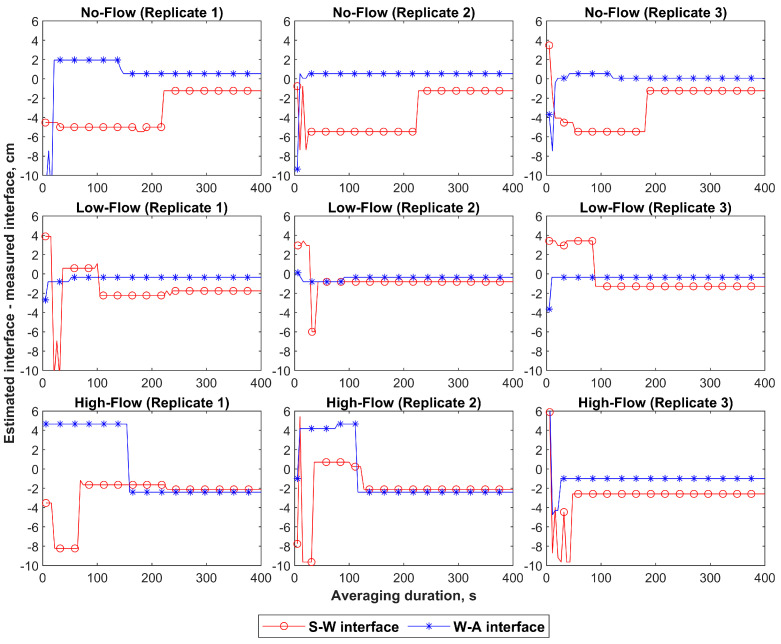
Errors in the detected interfaces derived from the mean temperature-increase signals over varying time intervals. The markers of the errors in the locations of the S-W and W-A interfaces are added every five data points for clarity purposes.

The impact of the measurement duration on the accuracy of the FO-DTS methods is shown in Figure 7. The mean and standard deviation of 211 ± 23 s, 124 ± 102 s, and 134 ± 90 s were required to obtain stable measurements of the S-W interface in the no-flow, low-flow, and high-flow conditions, respectively. A maximum period of 228 s was sufficient to accurately estimate the location of the S-W interface in all the studied cases except for the first replicate of the low-flow conditions, which required an average period of ~238 s (see Table 1).

On the other hand, shorter averaging durations of 99 ± 64 s, 51 ± 41 s, and 101 ± 67 s were required to obtain stable measurements of the W-A interface in the no-flow, low-flow, and high-flow conditions, respectively. The maximum average duration required to detect the W-A interface in all the tested conditions was 159 s. Therefore, a period of 238 s was sufficient to accurately estimate the location of the S-W and W-A interfaces in all tested conditions. 

## 4. Concluding Remarks

This technical note introduced a novel FO-DTS scour-monitoring device coupled with an automatic algorithm to detect the S-W and W-A interfaces. The testing of the FO-DTS scour-monitoring device in a laboratory flume demonstrated that the device can effectively detect the S-W and W-A interfaces under different flow conditions with mean absolute errors of 1.60 cm and 0.63 cm and maximum absolute errors of 2.59 cm and 2.41 cm, respectively. A measurement duration of <238 s was sufficient to obtain a stable measurement of the locations of the detected interfaces in all the tested conditions. It is worth noting that these results were achieved using a simple geometrical design and a straightforward non-calibrated detection algorithm. The accuracy of the FO-DTS scour-monitoring device also has the potential to be improved further by enhancing its geometrical design or by calibrating the detection algorithm.

In all the tested conditions, the section of the FO-DTS scour-monitoring device, which was surrounded by water, cooled relatively fast and almost reached ambient temperatures within approximately 200 s after the end of the heat pulse. The fast response of the water-surrounded device section highlights the potential of the FO-DTS scour-monitoring device to monitor active scour and water depth variations with high temporal resolutions of a few minutes.

The novel FO-DTS scour-monitoring device addresses many of the limitations of the existing scour-monitoring systems. Besides its capability of obtaining high-resolution measurements of the scour and water depths, it can also resist the harsh environmental conditions typically observed in actual streams due to the ruggedness of the used FO cable. In addition, the device can be used to monitor scour and water depth around piers and the abutments of existing as well as new bridges that are still under construction, as it can simply be attached to the sides of piers and abutments without the need to twine the FO cable around the actual structures. Furthermore, the device can measure high-resolution temperature profiles inside the sediment, along the water depth, and in the air, in addition to the scour and water depth, which can be useful for different applications. However, further research is needed to investigate the performance of the device in different types of sediment and field conditions, as well as to optimize the heating power and duration. 

## 5. Patents

The novel FO-DTS device discussed in this technical paper is currently protected by a provisional patent (application no. 63/416,705).

## Figures and Tables

**Table 1:** Errors in the estimated S-W and W-A interfaces and the minimum heating duration needed to obtain a stable measurement for the different tested conditions.

Test	S-W Error (cm)	S-W Minimum Heating Duration (s)	W-A Error (cm)	W-A Minimum Heating Duration (s)
No-flow (replicate 1)	−1.23	222	0.54	148
No-flow (replicate 2)	−1.23	227	0.54	26
No-flow (replicate 3)	−1.23	185	0.07	122
Low-flow (replicate 1)	−1.76	238	−0.35	53
Low-flow (replicate 2)	−0.82	43	−0.35	91
Low-flow (replicate 3)	−1.29	90	−0.35	10
High-flow (replicate 1)	−2.11	228	−2.41	159
High-flow (replicate 2)	−2.11	127	−2.41	116
High-flow (replicate 3)	−2.59	48	−0.99	27

## Data Availability

The data presented in this study are openly available online at FigShare https://doi.org/10.6084/m9.figshare.22558216 (accessed on 2 April 2023).
